# A study on the mechanism of Bushen Kaiqiao Formula in modulating microglial activation to alleviate neuroinflammation in ADHD

**DOI:** 10.1016/j.bbrep.2025.102211

**Published:** 2025-08-18

**Authors:** Jiaqi Zhang, Ruxin Sun, Yu Xiong, Yuting Yang, Jing Wang, Kanglin Zhu, Xinqiang Ni, Min Huang

**Affiliations:** a Department of Neurology, The Seventh Affiliated Hospital, Sun Yat-sen University, Shenzhen, China; b The Fourth Clinical Medical College, Guangzhou University of Chinese Medicine, Shenzhen, China

**Keywords:** Attention deficit hyperactivity disorder, Bushen Kaiqiao Formula, Neuroinflammation, Microglial activation, NF-κB pathway

## Abstract

**Background:**

Growing evidence suggests that dysregulated microglial activation and neuroinflammation in the prefrontal cortex may underlie the pathophysiology of Attention Deficit Hyperactivity Disorder. Utilizing spontaneously hypertensive rats—a well-validated animal model of ADHD—this study aimed to characterize ADHD-like behavioral phenotypes and prefrontal cortical neuronal development and elucidate the neuroimmunological mechanisms through which the Bushen Kaiqiao Formula (BSKQF) exerts its therapeutic effects by modulating microglial activation states.

**Methods:**

Ultra-high-performance liquid chromatography coupled with a tandem mass spectrometer (UHPLC-MS/MS) was used to qualitatively analyze the chemical composition of Bushen Kaiqiao Formula, and network pharmacology was applied to predict its potential therapeutic targets and ADHD-related pathways ADHD-like behaviors, including hyperactivity, impulsivity, spatial memory, and attention, were assessed using the Open Field Test, Elevated Plus Maze, and Y-Maze Test at weeks 2 and 4. Prefrontal cortical neuronal morphology and dendritic spine density were evaluated using Nissl staining and Golgi-Cox staining, respectively. Microglial activation (Iba-1+/iNOS + immunofluorescence), inflammatory cytokine levels (IL-1β and IL-6 by ELISA), and blood-brain barrier integrity (transmission electron microscopy) were examined. In vitro, primary prefrontal cortical neurons and microglia were isolated from neonatal rats and treated with BSKQF-containing serum at different concentrations, with Methylphenidate (MPH) as a control. An oxygen-glucose deprivation/reoxygenation (OGD/R) model was established to mimic neuroinflammatory conditions with inflammatory signaling pathways assessed.

**Results:**

UHPLC-MS/MS identified key bioactive components of Bushen Kaiqiao Formula. Network pharmacology analysis suggested that BSKQF may ameliorate ADHD-related behavioral deficits by inhibiting the NF-κB signaling pathway. Behavioral assessments demonstrated dose- and time-dependent effects. BSKQF significantly reduced neuronal loss, promoted synaptic plasticity, reduced the proportion of activated microglia (iNOS+/Iba-1+), and lowered IL-1β and IL-6 levels. Additionally, ultrastructural examination indicated restored blood-brain barrier integrity and reduced perivascular edema. In vitro, BSKQF-containing serum suppressed NF-κB pathway activation (p–NF–κB-p65, p-IκBα) and inflammasome-related proteins (NLRP3, caspase-1).

**Conclusion:**

BSKQF effectively alleviates ADHD-like behaviors in SHR rats by inhibiting prefrontal microglial activation and neuroinflammation, reducing neuronal apoptosis, restoring synaptic plasticity, and preserving blood-brain barrier integrity.

## Highlights

• BSKQF ameliorates ADHD-like behaviors by modulating prefrontal microglial activation.
• Chemical composition and potential molecular targets of BSKQF were identified via UPLC-MS and network pharmacology.
• BSKQF restored synaptic plasticity and reduced neuronal apoptosis in SHR rats and microglia-neuron co-culture models.
• In vitro, BSKQF-containing serum suppressed inflammatory signaling under OGD/R conditions.
• This study provides novel neuroimmune insights into the therapeutic potential of traditional Chinese medicine for ADHD.

## 1 Introduction

Attention deficit hyperactivity disorder (ADHD) is a common neurodevelopmental disorder characterized by inattention, hyperactivity, and impulsivity, affecting approximately 7 % of children and 5.6 % of adolescents [[1], [2], [3]], with around 15 % of patients continuing to meet diagnostic criteria beyond the age of 25 [[4], [5], [6]].

The etiology of ADHD involves a complex interplay of genetic and environmental factors [[7], [8], [9]]. Among various environmental contributors, epidemiological analyses have highlighted the role of hypoxia-ischemia, suggesting that oxygen-glucose deprivation (OGD) plays a critical role in such neurodevelopmental disorders [10]. Microglia are the primary immune cells of the central nervous system Its activation can lead to metabolic reprogramming by shifting from oxidative phosphorylation to glycolysis, which demands of acute inflammation [11,12]. OGD can lead to aberrant activation of microglia, which in turn alters the microenvironment of prefrontal cortical neurons, contributing to neuronal apoptosis [13]. Neuroimaging studies have revealed structural abnormalities in the prefrontal cortex of individuals with ADHD, which are associated with deficits in executive function and impulse control [14]. Moreover, elevated levels of proinflammatory cytokines in the serum of ADHD patients have been positively correlated with symptom severity, indicating that ADHD may be a highly inflammation- and immunity-associated disorder [15,16]. Although pharmacological treatments such as methylphenidate are widely used, their efficacy is often limited by side effects and risk of dependence [17]. In contrast, traditional Chinese medicine (TCM), with its holistic and multi-target therapeutic strategies, has shown potential for symptom alleviation and reduced adverse effects in ADHD management [18,19].

Recent studies have reported that herbal formulations, such as Dihuangyinzi and Anshen Dingzhi Ling, exhibit neuroprotective and anti-inflammatory effects in preclinical models of neuropsychiatric disorders [[20], [21], [22]]. Building upon these findings and decades of empirical clinical experience, we developed a novel empirical TCM formula, the Bushen Kaiqiao Formula (BSKQF), for the treatment of ADHD. BSKQF is composed of Rehmanniae Radix Praeparata, Plastrum Testudinis, Fossilia Ossis Mastodi, Polygalae Radix Praeparata, Acori Tatarinowii Rhizoma, Poria, Cinnamomi Ramulus, Paeoniae Radix Alba Praeparata, Bupleuri Radix Praeparata cum Aceto , Scutellariae Radix, Schisandrae Fructus Praeparata cum Aceto, Ophiopogonis Radix, and Glycyrrhizae Radix et Rhizoma Praeparata cum Melle. Previous studies suggest that Rehmanniae Radix Praeparata can inhibit ADHD-like behaviors by promoting hippocampal development [23], while Acori Tatarinowii Rhizoma may modulate monoaminergic neurotransmission to improve symptoms [24]. However, it remains unclear whether BSKQF can attenuate microglial overactivation and associated neuroinflammation, thereby improving ADHD-related behaviors via a multi-target mechanism.

To address this knowledge gap, we employed UHPLC-MS/MS to identify the major chemical constituents of BSKQF, and utilized network pharmacology to predict its putative targets and relevant signaling pathways. We further investigated its therapeutic efficacy and underlying mechanisms by using spontaneously hypertensive rats (SHRs) and an oxygen-glucose deprivation/reoxygenation (OGD/R) model in vivo and vitro.

## 2 Materials and methods

### 2.1 Animals

Sixty SPF-grade male SHR rats and ten male WKY rats, all three weeks old, were purchased from Zhongke Industrial Holdings (Shenzhen) Co., Ltd. (Shenzhen, China, Certificate No. SYXK (Yue) 2021-0263). All animals were housed in a temperature- and humidity-controlled environment (25 ± 1 °C) under a 12-h light/12-h dark cycle. Water and standardized rat food were provided ad libitum. The animal experiment was approved by the Laboratory Animal Ethics Committee of The Second People's Hospital of Shenzhen and conducted in strict accordance with the Guide for the Care and Use of Laboratory Animals issued by the National Institutes of Health. All efforts were made to minimize the number of animals used and to reduce their distress. Prior to gavage administration, animals were acclimatized and fed for one week.

### 2.2 Preparation of BSKQF extract

The Bushen Kaiqiao Formula (BSKQF) consists of Rehmanniae Radix Praeparata (15 g), Polygalae Radix Praeparata (10 g), Acorus Tatarinowii Rhizoma (10 g), Poria (10 g), Cinnamomi Ramulus (6 g), Paeoniae Radix Alba Praeparata (15 g), Bupleuri Radix Vinegar-Processed (6 g), Scutellariae Radix (6 g), Schisandrae Chinensis Fructus Vinegar-Processed (5 g), Ophiopogonis Radix (10 g), and Glycyrrhizae Radix et Rhizoma Praeparata (5 g). All herbal materials were purchased from Beijing TongRenTang Pharmacy (Beijing, China). The herbal ingredients were obtained from Tongrentang (Jinzhong Branch, Beijing, China). The herbs were first soaked in distilled water (1:10, w/v) for 30 min, then brought to a boil and simmered over low heat for 30 min. The resulting decoction was collected, and the extraction process was repeated with fresh distilled water under the same conditions. The two extracts were then combined and concentrated to obtain a stock solution.

### 2.3 Ultra-high-performance liquid chromatography coupled with a tandem mass spectrometer (UHPLC-MS/MS)

Approximately 0.2 g of lyophilized powder was dissolved in 5 mL of methanol–water (50:50, v/v), ultrasonicated, centrifuged, and filtered through a 0.22 μm membrane before injection. The analysis was performed using a UHPLC system(Waters Acquity, USA) coupled with a Xevo G2 Q-TOF mass spectrometer (Waters, USA). Chromatography was conducted on a BEH C18 column (100 mm × 2.1 mm, 1.7 μm) at 50 °C. The mobile phase consisted of acetonitrile (A) and 0.1 % formic acid aqueous solution (B). A gradient elution was employed with a flow rate of 0.4 mL/min: 0–15 min, 5–25 % A; 15–30 min, 25–70 % A; 30–35 min, 70–100 % A. The injection volume was 30 μL. Mass spectrometry was performed in both positive and negative ion modes with data acquisition in TOF-MSe mode (*m/z* 50–1300). The capillary voltage was set at ±3.0 kV, and the cone voltage was 40 V.

### 2.4 Network pharmacology analysis

ADME data for the drug components were obtained from the SWISSADME website using the SMILES numbers of the compounds, and the compounds were screened based on the following criteria: molecular weight (MW) ≤ 500, hydrogen bond donors (Hdon) ≤ 5, hydrogen bond acceptors (HAcc) ≤ 10, LogP ≤5, and gastrointestinal absorption (GI absorption) classified as high. Additionally, at least three of the following conditions were met: Lipinski violations = 0, Ghose violations = 0, Veber violations = 0, Egan violations = 0, and Muegge violations = 0. Compound targets were predicted by inputting the SMILES numbers into the SwissTargetPrediction database, with targets having a probability greater than 0 selected, and duplicates removed. ADHD-related genes were retrieved from GeneCards, CTD and OMIM databases, and the search results of the databases were summarized and duplicates were removed. The network of predicted targets and ADHD-related genes were constructed using Cytoscape. The potential targets for traditional Chinese medicine treatment of the disease were imported into STRING database to construct the protein-protein interaction (PPI) network, which was visualized in Cytoscape to obtain the correlation of these genes. GO and KEGG pathway analyses were performed using the DAVID database.

### 2.5 Administration of RRP and MPH

The experiment included six groups: normal control (WKY), model (SHR), low-, medium-, and high-dose BSKQF treatment groups (LOW, MID, HIGH), and a positive control (MPH). Sixty SHR rats were randomly assigned to the SHR, LOW, MID, HIGH, and MPH groups (N = 10 per group). Although there is currently no standardized formula specifically designed for dose conversion of traditional Chinese herbal compound formulas, we referred to the commonly used body surface area (BSA) normalization method to determine the standard dose: $Animal\;dose\left(\frac{g}{kg}\right)=Human\;dose\left(\frac{g}{kg}\right)\times \frac{{Km}_{Human}}{{Km}_{Animal}}$ [25]. Based on this calculation, the equivalent clinical dose was estimated to be 30.49 g/kg, which was designated as the medium-dose group. The low- and high-dose groups were set at 0.5 × and 2 × of the medium dose, respectively. Methylphenidate (MPH) was administered at a dose of 2 mg/kg/day. All animals received intragastric administration twice daily (1 mL/100 g body weight) for a duration of 4 weeks. The compound formula dosage was determined based on clinical equivalents and prior data, with standard, low (0.5 × ), and high (2 × ) doses set at 30.49 g/kg. MPH was administered at 2 mg/kg/day. All animals received intragastric administration (1 mL/100 g body weight) twice daily for four weeks. The WKY and SHR groups received an equal volume of CMC-Na. Behavioral tests (EPM, OFT, Y-maze) were conducted at weeks 0, 2, and 4, 30 min after the first dose. Brain tissue was collected post-experiment for analysis or stored at −80 °C.

### 2.6 Open Field Test(OFT)

The open field test was conducted to evaluate spontaneous locomotor activity and exploratory behavior in rodents [26]. The open field test was conducted in a square arena (25 cm × 25 cm grids) with a defined center area. Each rat was placed in the center and allowed to explore for 5 min. The system recorded total distance moved, average speed, center entries, and center movement distance, along with movement trajectories and heatmaps. The arena was cleaned with 75 % ethanol after each trial. Tests were performed before, at 2 weeks, and at 4 weeks post-administration to assess treatment effects.

### 2.7 Elevated Plus Maze (EPM) test

The elevated plus maze (EPM) test was conducted to assess impulsivity and anxiety-related behavior in rodents [[27], [28], [29]]. The apparatus consisted of two open arms and two closed arms connected by a central platform. Before testing, rats were acclimated to the experimental environment for 30 min. During the test, each rat was gently placed on the central platform and allowed to explore freely for 5 min. The behavioral analysis system recorded the total movement distance (cm), the number of entries into the open arms, and the time spent in the open arms and closed arms (s). After each trial, the maze was cleaned with 75 % ethanol to remove feces and residual odors, ensuring the accuracy of subsequent tests.

### 2.8 Y maze

The Y-maze test was conducted to assess spatial working memory and attentional function in rodents [30,31]. The apparatus consisted of three arms of equal length and width, arranged at a 120° angle. Before testing, animals were allowed to explore the maze freely for 10 min for habituation. During the test, each rat was gently placed in the central area, facing the entrance of one arm, and allowed to explore freely for 8 min. The video tracking system recorded the total movement distance (cm), average speed (cm/s), and spontaneous alternation behavior (consecutive entries into three different arms). The alternation percentage was calculated as [% Alternation = (actual alternations/(total entries − 2)) × 100]. After each trial, the maze was thoroughly cleaned with 75 % ethanol to eliminate residual odors and ensure consistent experimental conditions.

### 2.9 Sample collection

After behavioral testing, rats (N = 10 per group) were fasted overnight and anesthetized with isoflurane (RWD, R510-22-10). For brain tissue staining, four rats from each group were randomly selected. These rats were transcardially perfused with 100 mM cold phosphate-buffered saline (PBS, pH 7.4), followed by 4 % cold paraformaldehyde (PFA) until fixed limb convulsions were observed, indicating successful perfusion. The brains were immediately extracted, post-fixed in 4 % PFA at 4 °C for 24 h, and subsequently embedded in paraffin. Coronal brain sections (5 μm thick) were prepared using a standard microtome (Leica, Wetzlar, Germany) and analyzed with various staining techniques, including immunofluorescence and Nissl staining. For the remaining rats in each group, transcardial perfusion with cold PBS was performed, followed by rapid extraction of the entire brain. The left and right prefrontal cortices were dissected on ice. For transmission electron microscopy (TEM) analysis, 1-mm^3^ tissue blocks from the left prefrontal cortex were isolated and fixed (*N* = 3 rats/group). The right prefrontal cortex of each rat was immersed in Golgi-Cox staining solution (*N* = 3 rats/group). For enzyme-linked immunosorbent assay (ELISA) analysis, the prefrontal cortex was rapidly dissected on a cold platform, weighed, and immediately frozen in liquid nitrogen (*N* = 3 rats/group).

### 2.10 Nissl staining

The prefrontal cortex tissues were placed in 4 % paraformaldehyde (Servicebio, cat: G1101) for 24 h and subsequently embedded in paraffin. Paraffin sections were cut to a thickness of 5 μm for Nissl staining. To prevent the sections from detaching, they were baked at 60 °C for 2 h in a drying oven. For the staining procedure, the sections were dewaxed, rehydrated, and then immersed in a 0.1 % cresyl violet staining solution for 12 min at 37 °C. Following the staining, the sections were sequentially immersed in 70 % ethanol (AC) for 30 s, 80 % ethanol for 30 s, 95 % ethanol I for 30 s, 95 % ethanol II for 30 s, ethanol I for 5 s, and ethanol II for 5 s. Afterward, the sections were dried and immersed in Dibenzoylmethane (DMB) I for 2 min and DMB II for 2 min. Finally, the sections were mounted and sealed with neutral balsam. Three slices were analyzed for each group (*N* = 3 rats/group).

### 2.11 Golgi-Cox staining

Golgi-Cox staining is a neuronal staining technique used to assess synaptic plasticity. Fresh brain tissue was obtained from three rats per group and washed with PBS. The tissue was fully immersed in Golgi-Cox staining solution (Servicebio, Wuhan, China) and stored in a cool, dark place for 14 days. After staining, the tissue blocks were removed, dehydrated, and treated with a developer for 45 min. Subsequently, the samples were dehydrated, frozen, sectioned, and mounted with glycerin gelatin. Images of neurons and dendritic spines in the prefrontal cortex were captured using an upright electron microscope (NIKON, Japan) and CaseViewer software. The images were then analyzed using ImageJ software and GraphPad Prism 8.0. For each group (*N* = 3 rats/group), a total of three sections were analyzed.

### 2.12 Immunofluorescence staining

Paraffin sections were equilibrated to room temperature for 30 min. The sections were then washed with PBS for 5 min, repeated three times. To block non-specific binding, 5 % goat serum was applied, and the sections were incubated at room temperature for 30 min. After removing the blocking buffer, a mixture of primary antibodies was added, including mouse anti-Iba-1 (1:500) (Servicebio, GB15105-100)and rabbit anti-iNOS (1:200)(Servicebio, GB11119-100), followed by overnight incubation at 4 °C in the dark. After primary antibody incubation, the sections were washed with PBS for 5 min, repeated three times. Subsequently, a mixture of secondary antibodies was applied, including FITC-conjugated goat anti-rabbit IgG (1:500)(Servicebio, GB25303) and Cy3-conjugated goat anti-mouse IgG (1:500)(Servicebio, GB21301). The sections were then incubated in a humidified chamber at 37 °C for 1 h in the dark. After incubation, the cell nuclei were counterstained with DAPI at room temperature for 5 min. The number of Iba-1 and iNOS double-labeled positive cells in the prefrontal cortex region was counted using ImageJ software.

### 2.13 Cytokine measurements by Enzyme-Linked Immunosorbent Assay (ELISA)

The brain tissues were collected, homogenized, and the supernatant was extracted for analysis. Cytokine levels, including IL-6 and IL-1β, were quantified using an ELISA kit (Servicebio, GER0001; Elabscience, E-EL-R0012).Briefly, a 96-well plate was coated with a primary antibody, and 100 μL of either sample or standard solution was added to each well. Subsequently, 100 μL of biotinylated detection antibody was introduced, followed by incubation at room temperature for 1 h. After incubation, the wells were aspirated and washed with PBS. Then, 100 μL of streptavidin complex reagent was added and incubated at room temperature for another hour. For colorimetric detection, 90 μL of TMB substrate solution was added to each well and incubated at room temperature for 20 min. The reaction was terminated by adding 50 μL of stop solution. Absorbance was measured at 450 nm using a multimode microplate reader. A standard curve was generated based on the optical density (OD) values of the standard reagent, and cytokine concentrations were determined accordingly. (N = 3 rats/group).

### 2.14 Transmission Electron Microscopy (TEM)

The prefrontal cortex tissue was dissected and rinsed three times with 0.1 M PBS. The samples were then fixed in 1 % osmium tetroxide in 0.1 M PBS for 2 h, followed by three additional PBS washes. Dehydration was performed sequentially using graded ethanol solutions (50 %, 70 %, 80 %, 90 %, 95 %, and 100 %) and acetone (100 %) for 15 min at each step. The tissues were then infiltrated with a 1:1 mixture of epoxy resin and acetone for 2–4 h at room temperature. After infiltration, the samples were embedded in molds and incubated at 37 °C overnight, followed by polymerization at 60 °C for 48 h. Ultrathin sections were double-stained with 2.5 % uranyl acetate and Reynolds’ lead citrate for 15 min each. The sections were air-dried overnight at room temperature. Imaging and analysis were conducted using transmission electron microscopy (*N* = 3 rats/group).

### 2.15 Cell culture

As previously documented, primary microglia were extracted from 1-day-old Sprague-Dawley rats. Once the mixed glial cultures had reached confluence, the separation of microglia from astrocyte monolayers was achieved through the application of mild shaking at 200 rpm (GS-20, MIULAB) at 37 °C for 4 h. The microglia were maintained in DMEM/F12 (Gibco, USA), with 10 % FBS (Gibco, USA) and 1 % penicillin/streptomycin (Gibco, USA). To establish in vitro OGD/R model, the medium of microglia was replaced by DMEM without glucose (Gibco, USA) and the cells were subjected to a gas mixture of 95 % N2 and 5 % CO2. To prepare drug-containing serum, Sprague-Dawley (SD) rats were randomly divided into a drug serum group and a control group. Rats in the drug serum group received intragastric administration of a high dose of the test formulation twice daily for 7 consecutive days, while those in the control group were administered an equal volume of normal saline. Thirty minutes after the final administration, rats were anesthetized with isoflurane, and blood samples were collected from the abdominal aorta. The samples were allowed to stand at room temperature for 2 h and then centrifuged at 3000 rpm for 15 min at room temperature. The supernatant serum was collected, heat-inactivated in a 56 °C water bath for 30 min, filtered through a 0.22 μm filter, aliquoted, and stored at −80 °C until use. After OGD/R for 4 h, the primary microglia were treated with drug-containing serum at final concentrations of 5 %, 10 %, and 20 % to simulate low, medium, and high doses for 24 h, respectively. The MPH group was treated with complete culture medium containing methylphenidate hydrochloride as positive control. Control microglia were cultured in normal condition. After different treatment, total proteins were extracted using RIPA containing 1 % PMSF and 1 % phosphatase inhibitor. Proteins were separated in SDS-PAGE and transferred onto a PVDF membrane. After a quick blockade, the membranes were incubated with primary antibody, followed by HRP-conjugated secondary antibodies. ECL was used to detect protein contents.

### 2.16 Image analysis and statistical analysis

Statistical analyses were performed using GraphPad Prism 8.0.2. Optical density in Western blot and positive cell counts in fluorescence images were quantified using ImageJ-Fiji. Data are presented as mean ± standard deviation (SD). Normality was assessed using the Normality and Lognormality test, and variance homogeneity was evaluated with the Brown–Forsythe test. Group comparisons were conducted via one-way or two-way ANOVA followed by Tukey's post hoc test, with significance set at p < 0.05.

## 3 Results

### 3.1 UHPLC-MS analysis of BSKQF extract

**Fig. 1:**
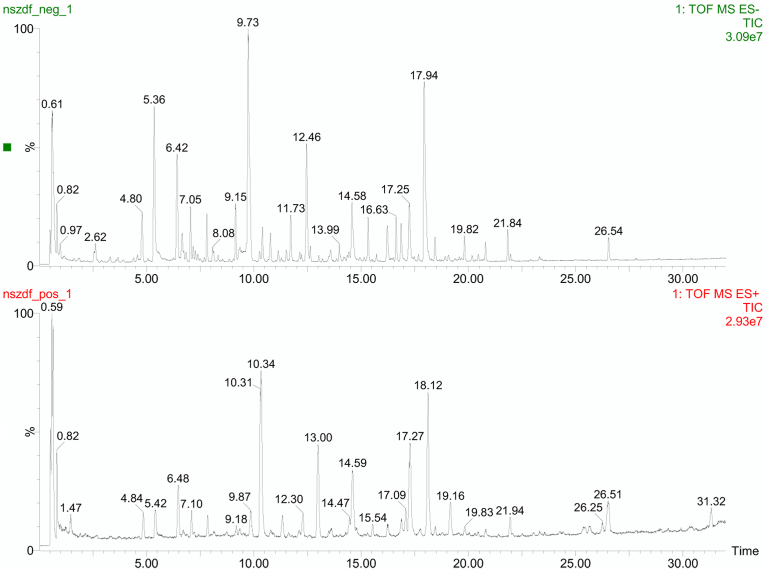
BSKQF were shown by total ion chromatography in negative (upper) and positive (lower) ion modes.

**Fig. 2:**
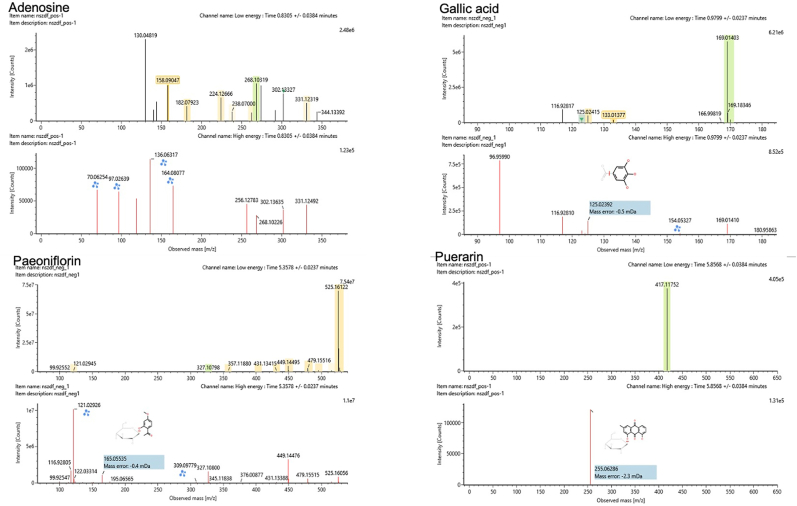
UHPLC-MS characteristics of BSKQF extract The aqueous extract of Bushen Kaiqiao Formula (BSKQF) contains the following active compounds: Adenosine, Gallic acid, Paeoniflorin, Puerarin.

Using UHPLC-MS, we successfully analyzed the chemical composition of the Bushen Kaiqiao formula, as shown in Fig. 1. After removing redundant data, several distinct ion peaks were identified as major compounds. The molecular formulas of these compounds were determined based on high-resolution mass-to-charge ratios (*m/z*), and candidate structures were initially inferred from literature and public databases. These structures were further confirmed by MS fragmentation analysis. In total, 138 components were identified from the formula. As illustrated in Fig. 2, several compounds, including adenosine, gallic acid, paeoniflorin, and puerarin, were successfully identified. These representative compounds—adenosine, gallic acid, puerarin, and paeoniflorin—share common anti-inflammatory and immunomodulatory properties, primarily through the regulation of microglial activation and cytokine release, thus contributing to the improvement of cognitive and behavioral dysfunctions associated with neuroinflammatory conditions such as ADHD [[32], [33], [34], [35], [36]].

### 3.2 Network pharmacology analysis of BSKQF

**Fig. 3:**
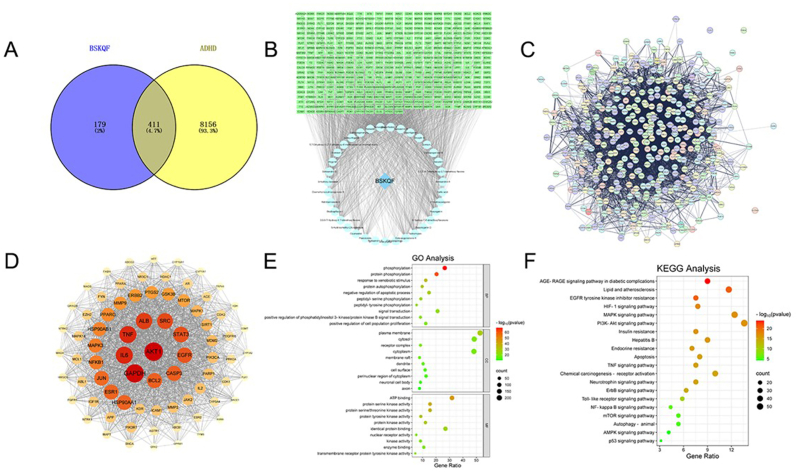
Mass-spectrometry based network pharmacology analysis of BSKQF (A) Drug-disease intersection targets; (B) Drug-compound-target network; (C, D) Protein-protein interaction (PPI) network; (E) Gene Ontology (GO) functional enrichment analysis; (F) Kyoto Encyclopedia of Genes and Genomes (KEGG) pathway enrichment analysis.

Network pharmacology analysis was performed to identify potential functional targets of the BSKQF compounds detected by UHPLC-MS. After removing duplicates, 590 compound-related targets and 8567 ADHD-related targets were identified. The obtained genes were standardized using the UniProt database. The intersection of drug and disease targets yielded 411 overlapping genes (Fig. 3-AB). These genes were imported into the STRING database) for protein-protein interaction (PPI) analysis, with the species set to *Homo sapiens* and a confidence threshold of 0.4. The PPI network was saved in TSV format and visualized using Cytoscape 3.8.2, where node size and color reflect degree values (Fig. 3-CD). GO functional annotation(Fig. 3-E) and KEGG pathway analysis(Fig. 3-F) were conducted using the DAVID database, and the results were visualized. Notably, KEGG analysis revealed that BSKQF primarily modulates the NF-κB pathway, a key neuroinflammatory signaling pathway of interest.

### 3.3 Effects of BSKQF on locomotor activity and hyperactivity in the open field test in ADHD model rats

**Fig. 4:**
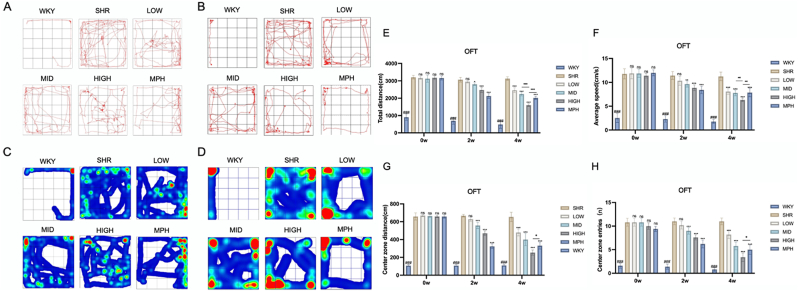
Representative track map of the behavioral experiments in various groups of rats (A, C) Representative trajectory and heatmap of the open field test (OFT) in each group after 2 weeks of gavage; (B, D) Representative trajectory and heatmap of the OFT after 4 weeks of gavage; (E) Total travel distance; (F) Average speed; (G) Distance traveled in the central zone; (H) Number of central zone entries. All data are presented as mean ± SD. Comparisons between BSKQF-treated groups and the SHR group were analyzed using one-way ANOVA followed by Tukey's multiple comparison test. ∗*p* < 0.05, ∗∗*p* < 0.01, ∗∗∗*p* < 0.001, Comparisons between WKY groups and the SHR group were analyzed using one-way ANOVA followed by Tukey's multiple comparison test, ###*p* < 0.001.

The open field test revealed that, compared to the WKY group, the SHR group exhibited significantly increased total travel distance(Fig. 4-E) (*P* < 0.001), average speed(Fig. 4-B) (*P* < 0.001), number of center entries(Fig. 4-G) (*P* < 0.001), and center travel distance(Fig. 4-H) (*P* < 0.001). After two weeks of treatment, the MPH and medium- and high-dose BSKQF groups showed significant reductions in these parameters compared to the SHR group (*P* < 0.001), whereas the low-dose group showed no significant changes. After four weeks, the low-dose group also exhibited reductions(*P* < 0.001), with the high-dose group showing more pronounced effects than the MPH group (*P* < 0.05). Moreover, the reductions in total travel distance and average speed were less pronounced in the high-dose group than in the medium-dose group (*P* < 0.01), suggesting a dose-dependent effect. These findings indicate that BSKQF significantly alleviates hyperactivity and impulsivity in ADHD model rats in a dose- and time-dependent manner.

### 3.4 Effects of BSKQF on impulsivity and anxiety-like behavior in the elevated plus maze test in ADHD model rats

**Fig. 5:**
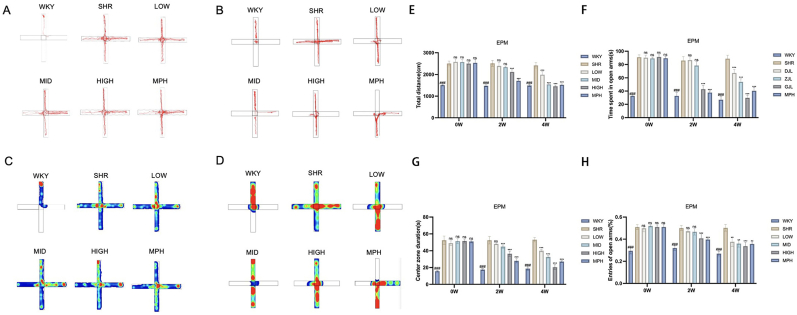
Representative trajectory maps of the elevated plus maze (EPM) test in different groups of rats. (A, C) Representative trajectory and heatmap of the EPM test after 2 weeks of gavage; (B, D) Representative trajectory and heatmap of the EPM test after 4 weeks of gavage; (E) Total travel distance; (F) Time spent in the open arms; (G) Time spent in the central zone; (H) Number of open-arm entries. All data are presented as mean ± SD. Comparisons between BSKQF-treated groups and the SHR group were analyzed using one-way ANOVA followed by Tukey's multiple comparison test. ∗*p* < 0.05, ∗∗*p* < 0.01, ∗∗∗p < 0.001, Comparisons between WKY groups and the SHR group were analyzed using one-way ANOVA followed by Tukey's multiple comparison test, ###*p* < 0.001.

The elevated plus maze test showed that the SHR group exhibited more total distance(Fig. 5-E), more open-arm entries(Fig. 5-H), and longer time spent in the open arms(Fig. 5-F) and center zone(Fig. 5-G) compared to the WKY group (*P* < 0.001). After two weeks of treatment, the MPH and high-dose BSKQF groups showed significant reductions in these parameters compared to the SHR group (*P* < 0.001), while the low- and medium-dose groups showed no significant changes. After four weeks, the low- and medium-dose groups also exhibited significant reductions in all measures (*P* < 0.01). These findings indicate that BSKQF effectively ameliorates impulsivity and anxiety-related behaviors in ADHD model rats in a dose- and time-dependent manner.

### 3.5 Effects of BSKQF on spatial working memory and attention in the Y-maze test in ADHD model rats

**Fig. 6:**
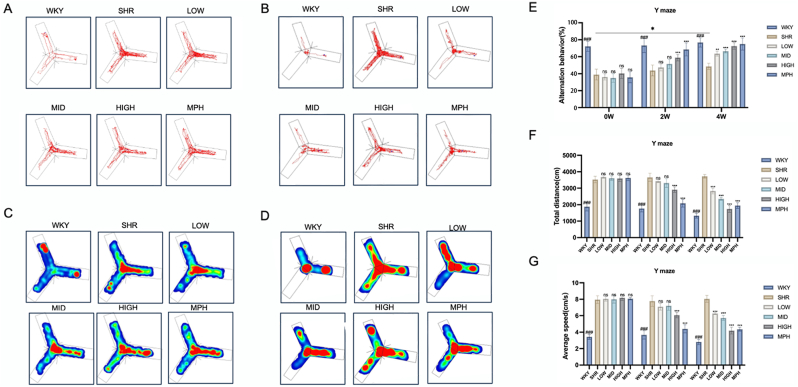
Representative track map of the behavioral experiments in various groups of rats (A, C) Representative trajectory and heatmap of the Y-maze test after 2 weeks of gavage; (B, D) Representative trajectory and heatmap of the Y-maze test after 4 weeks of gavage; (E) Spontaneous alternation rate; (F) Total travel distance; (G) Average movement time. All data are presented as mean ± SD. Comparisons between BSKQF-treated groups and the SHR group were analyzed using one-way ANOVA followed by Tukey's multiple comparison test. ∗*p* < 0.05, ∗∗*p* < 0.01, ∗∗∗*p* < 0.001, Comparisons between WKY groups and the SHR group were analyzed using one-way ANOVA followed by Tukey's multiple comparison test, ###*p* < 0.001.

The Y-maze test revealed that the SHR group exhibited a lower spontaneous alternation rate Fig. 6-E), greater total travel distance(Fig. 6-F), and higher average speed(Fig. 6-G) compared to the WKY group (*P* < 0.001). After two weeks of treatment, the MPH and high-dose BSKQF groups showed a significant increase in spontaneous alternation rate and a decrease in total travel distance and speed compared to the SHR group (*P* < 0.001), while no significant changes were observed in the low- and medium-dose groups. After four weeks, the low- and medium-dose groups also exhibited improved spontaneous alternation rates and reduced travel distance and speed (*P* < 0.001). Notably, untreated SHR rats also showed a slight increase in spontaneous alternation rate at four weeks(*P* < 0.05), possibly reflecting adaptation to the maze. These findings suggest that BSKQF significantly enhances spatial memory and attention in ADHD model rats in a dose- and time-dependent manner.

### 3.6 BSKQF suppresses prefrontal microglial activation and attenuates inflammation

**Fig. 7:**
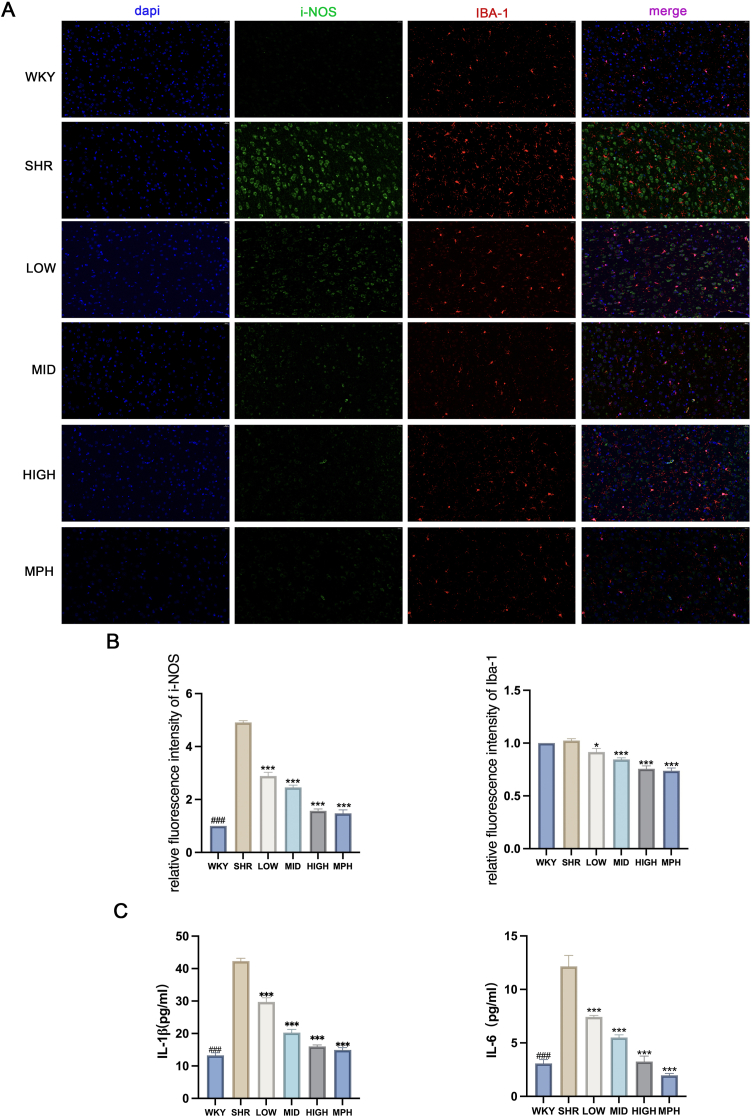
Prefrontal microglia activation and inflammatory factor levels (A) Immunofluorescence staining of microglia in the prefrontal cortex of each group. Iba-1^+^ cells (red), iNOS^+^ cells (green), and DAPI-stained nuclei (blue). Scale bar = 10 μm. (B) Quantification of immunofluorescence staining.All data are presented as mean ± SD. Comparisons between BSKQF-treated groups and the SHR group were analyzed using one-way ANOVA followed by Tukey's multiple comparison test. ∗*p* < 0.05, ∗∗*p* < 0.01 (##), ∗∗∗*p* < 0.001.

iNOS, a key marker of microglial activation under inflammatory conditions, and Iba-1, a microglial-specific calcium-binding protein, were used to assess microglial activation and function in Fig. 7-A(Scale bar = 20μm). As shown in Fig. 7-B, SHR rats exhibited excessive microglial activation in the prefrontal cortex, indicated by a significant increase in iNOS fluorescence intensity (*P* < 0.001) and elevated levels of pro-inflammatory cytokines IL-6 and IL-1β compared to the WKY group (*P* < 0.001). Both BSKQF and MPH treatment significantly reversed these changes (*P* < 0.001).

### 3.7 Effect of BSKQF on the blood-brain barrier in ADHD model rats

**Fig. 8:**
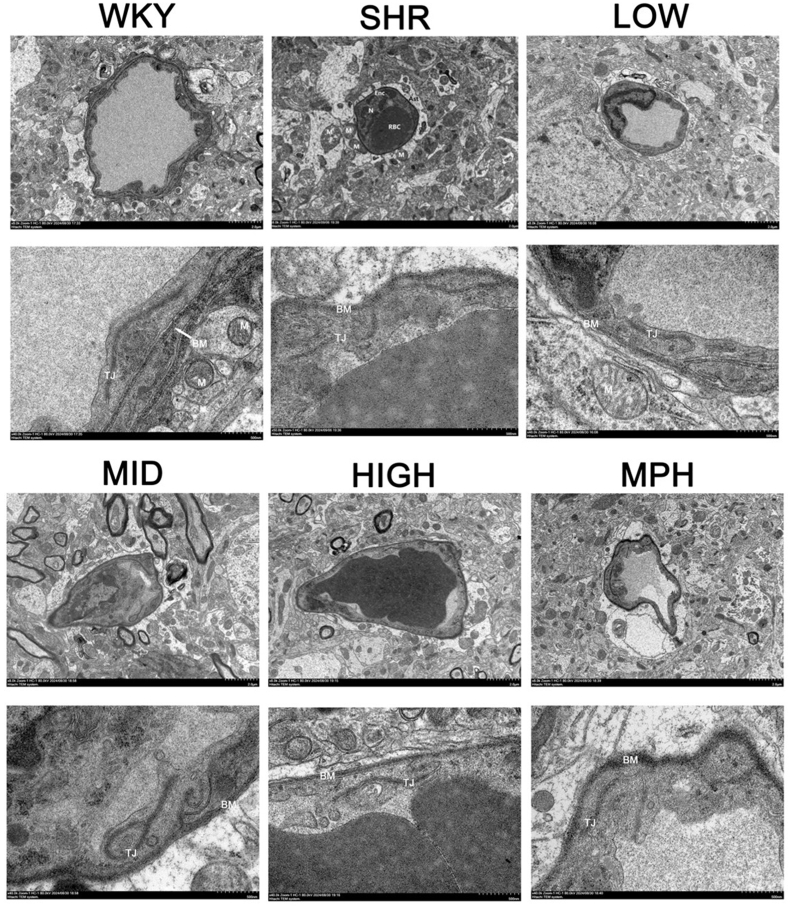
Ultrastructure of the blood-brain barrier in the prefrontal lobe.

To investigate whether SHR rats exhibit alterations in the blood-brain barrier (BBB)(Fig. 8, The scale bar represents 2 μm(up) or 500 nm(down) and is divided into 10 equal segments), we performed ultrastructural analysis using electron microscopy. Compared to the WKY group, SHR rats showed irregularly shaped endothelial cell nuclei with blurred nuclear membranes, tight junctions (TJs) with high electron density, and a disrupted basal membrane (BM) structure. Additionally, astrocytic end-feet (Ast) were edematous, with large areas of low electron density in the edema zone, swollen mitochondria (M), and a loss of matrix structure with fading and decreased cristae. After treatment with low, medium, and high doses of BSKQF, the BBB structure was significantly improved, as indicated by a clearer and continuous basal membrane, absence of noticeable edema in the surrounding parenchyma, and well-preserved endothelial cell structure with evenly distributed cell matrices and normal nuclei.

### 3.8 Effect of BSKQF on neuronal pathological morphology in ADHD model rats

**Fig. 9:**
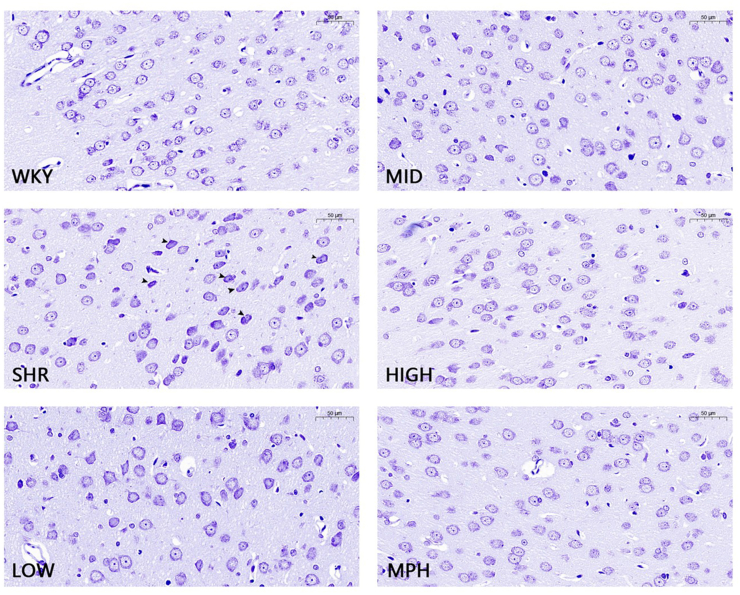
Nissl's staining analysis results.

Nissl staining showed significant neuronal pathology in SHR rats(Fig. 9, Scale bar = 50μm), including atrophy, shrinkage, nuclear fragmentation, and dissolution (black arrows). There was also a reduction in Nissl bodies and disordered neuronal arrangement. BSKQF treatment reversed these changes: the low-dose group showed partial shrinkage but improved nuclear integrity and Nissl body distribution; the medium-dose group further improved morphology; and the high-dose and MPH groups had better neuronal arrangement, approaching WKY controls. These results suggest BSKQF may protect neuronal structure and function.

### 3.9 Effect of BSKQF on dendritic spine density in the prefrontal cortex of ADHD model rats

**Fig. 10:**
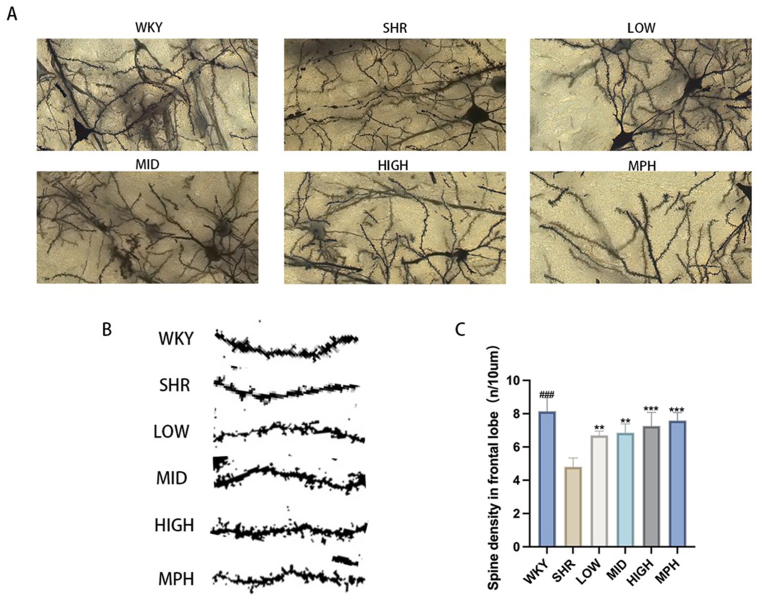
Glogi staining of prefrontal cortex neurons (A) Golgi staining of prefrontal cortex neurons in each group. (B) Quantification of dendritic spine density in prefrontal cortex neurons. All data are presented as mean ± SD. Comparisons between BSKQF-treated groups and the SHR group were analyzed using one-way ANOVA followed by Tukey's multiple comparison test. ∗∗p < 0.01, ∗∗∗p < 0.001; Comparisons between WKY groups and the SHR group were analyzed using one-way ANOVA followed by Tukey's multiple comparison test, ###p < 0.001.

Synaptic connections between neurons form neural circuits, which are fundamental to normal brain function. Dendritic spine density is a key indicator of synaptic plasticity and is closely related to learning and memory [37]. During synaptic plasticity, alterations in neuroinflammation can affect synaptic maturation, thereby influencing the formation and maturation of neural networks [38,39]. To assess dendritic spine density and morphology in prefrontal cortex neurons, we performed quantitative Golgi staining. Fig. 10-A shows representative micrographs of dendritic branches in the prefrontal cortex of rats(Scale bar = 10 μm). Fig. 10-B quantifies the number of spines per 10 μm of dendrite length. We observed a significant decrease in dendritic spine density in the SHR group compared to the WKY group(Fig. 10-C)(*P* < 0.001). BSKQF treatment at various concentrations significantly increased dendritic spine density in SHR rats (*P* < 0.01), with a dose-dependent trend. These findings suggest that BSKQF may restore neurogenesis in the prefrontal cortex of ADHD model rats, thereby repairing neural circuits.

### 3.10 BSKQF inhibits microglial inflammation pathways and alleviates inflammation

**Fig. 11:**
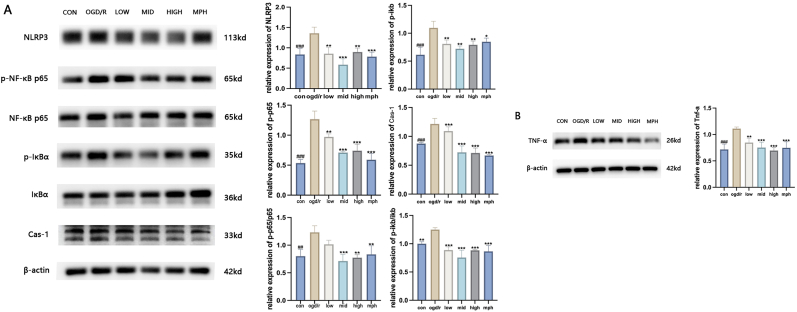
Effects of different concentrations of Kidney Bu Kai Fang on microglial inflammatory pathway after OGD/R treatment (A) Expression and statistical analysis of key proteins in the NLRP3/caspase-1 and NF-κB-p65 signaling pathways. (B) Expression of TNF-α. All data are presented as mean ± SD (n = 3 per group). Comparisons were performed using one-way ANOVA followed by Tukey's multiple comparison test. ∗∗p < 0.05, ∗∗∗p < 0.001, Comparisons between WKY groups and the SHR group were analyzed using one-way ANOVA followed by Tukey's multiple comparison test, ###p < 0.001.

Western blot analysis was performed to assess the expression levels of key proteins. Based on network pharmacology results, BSKQF is proposed to exert therapeutic effects in ADHD through modulation of the NF-κB signaling pathway. Thus, we quantitatively analyzed key NF-κB pathway proteins (including p–NF–κB-p65, NF-κB-p65, p-IκBα, and IκBα) in microglial cells following OGD/R model induction and treatment with different concentrations of drug-containing serum. Results showed that OGD/R treatment significantly increased the expression of p–NF–κB-p65 and p-IκBα, as well as the ratios of p–NF–κB-p65/NF-κB-p65 and p-IκBα/IκBα (Fig. 11-A) (*P* < 0.001), indicating phosphorylation of NF-κB-p65 and IκBα, thus enhancing NF-κB signaling transduction. BSKQF treatment inhibited this process, with the most significant inhibition observed in the medium-dose (10 % drug-containing serum) group.

Additionally, we assessed the activation of the NLRP3/caspase-1 inflammasome(Fig. 11-A) (*P* < 0.001), finding a trend consistent with NF-κB-p65 signaling. Further investigation revealed that all concentrations of BSKQF drug-containing serum suppressed TNF-α production(Fig. 11-B) (*P* < 0.001), with the high-dose group (20 %) showing superior suppression compared to the medium-dose group. This suggests that high-concentration drug-containing serum may enhance anti-inflammatory effects through alternative mechanisms. These results indicate that BSKQF may alleviate neuroinflammation by synergistically modulating both NF-κB and NLRP3 signaling pathways in microglia, thereby inhibiting the release of inflammatory cytokines.

## 4 Discussion

Attention Deficit Hyperactivity Disorder (ADHD) is a common neurodevelopmental disorder. Based on clinical observations and research, ADHD is considered a highly inflammatory and immune-related disease. Overactivated microglia contribute to behavioral defects, increasing susceptibility to ADHD [40]. Children with ADHD exhibit excessive region-specific microglial activation [41]. In recent years, the role of Traditional Chinese Medicine in ADHD treatment has gained increasing attention. Its formulations regulate neuroendocrine and immune-inflammatory responses through various pharmacological actions, helping to alleviate core symptoms of ADHD and reducing the risk of side effects from long-term use of psychotropic drugs [18,19,42]. Based on the pathogenesis of ADHD and the pharmacological effects of BSKQF, we hypothesize that BSKQF may be a safe and effective treatment for ADHD.

Through UHPLC-MS analysis of RRP extracts, several potential compounds were identified, including adenosine, gallic acid, paeoniflorin, and puerarin. Adenosine regulates attention deficits and hyperactivity in various diseases [32]. Gallic acid and puerarin inhibit microglial release of IL-6 and other inflammatory factors [33,34]. Paeoniflorin's anti-inflammatory and antioxidant effects not only upregulate monoamine neurotransmitter levels but also show significant efficacy in neurodegenerative diseases [35,36,43]. Furthermore, network pharmacology analysis revealed that BSKQF exerts its therapeutic effects on ADHD through multi-molecular, multi-target, and multi-pathway mechanisms, with NF-κB, an inflammation-related pathway, identified in the KEGG results.

In this study, behavioral tests, brain tissue staining, and ELISA were used to assess the behavioral and neuroinflammatory effects in male SHR rats. Behavioral experiments revealed that BSKQF exerts dose- and time-dependent effects. Although the onset of the medium- and low-dose treatments was slower than MPH, the effects were comparable over time. The open field test, which measures total distance traveled and number of entries into the center zone, showed that BSKQF and MPH significantly reduced hyperactive behaviors in SHR rats, with effects strengthening over time. The elevated plus-maze test demonstrated that WKY rats preferred the safe area, while SHR rats were more impulsive and less risk-averse, reflecting anxiety regulation deficits in SHR rats. Both BSKQF and MPH significantly improved these impulsive and anxiety regulation abnormalities. The spontaneous alternation rate, which reflects spatial memory and attention, indicated that both MPH and BSKQF enhanced SHR rats’ spatial learning and memory, with increased alternation rates as training progressed. In summary, BSKQF improved ADHD-like behaviors in young SHR rats by reducing spontaneous activity and impulsive behaviors, as well as improving learning and memory functions.

Reduced prefrontal cortex volume in ADHD is attributed to neuronal apoptosis and autophagy [44]. The brain's microenvironment plays a critical role in neuronal growth and development. Multiple studies suggest that intrauterine inflammation disrupts fetal neurodevelopment, with maternal inflammation programming the fetal central immune system via inflammatory responses and epigenetic mechanisms, leading to persistent epigenetic memory in microglia [[45], [46], [47]]. This results not only in neuronal apoptosis and autophagy but also in excessive pruning [48].

In our experiment, Iba-1/i-NOS dual immunofluorescence staining of the prefrontal cortex revealed abnormal microglial activation in SHR rats, accompanied by increased levels of pro-inflammatory cytokines IL-6 and IL-1β. BSKQF treatment reduced microglial activation, decreasing i–NOS–positive cells and alleviating prefrontal inflammation. Additionally, Nissl staining showed that BSKQF alleviated neuronal loss and distribution irregularity in the SHR prefrontal cortex. Golgi staining revealed a decrease in dendritic spine density in SHR rats, suggesting excessive dendritic spine pruning, which is associated with microglial overactivation. BSKQF treatment reversed this effect. TEM showed typical pathological changes in the BBB in the SHR group, which were significantly improved after BSKQF treatment, likely due to reduced central nervous system inflammation. Notably, there exists a bidirectional regulation between neuroinflammatory microenvironments and BBB integrity. BBB damage allows peripheral inflammatory factors to penetrate the central nervous system, exacerbating neuroinflammation and causing abnormal neuronal development. This provides new intervention strategies for ADHD treatment.

Despite the promising findings, this study has several limitations. First, BSKQF is a multi-component formulation with complex pharmacological effects, yet the study primarily focused on the neuroinflammation. Other potential mechanisms involved in the regulation of neurodevelopmental disorders were not thoroughly explored. Further studies should investigate alternative signaling pathways and molecular targets modulated by BSKQF. Second, while this study identified several bioactive compounds in BSKQF, it remains unclear which specific components exert the primary therapeutic effects. Future research should include monomeric compound studies to determine the individual and synergistic contributions of key constituents to ADHD treatment. Third, potential sex differences in ADHD pathophysiology and response to treatment were not considered. Given the well-documented sex-specific differences in ADHD incidence, symptomatology, and neuroinflammatory responses, future studies should examine whether BSKQF exhibits differential efficacy between male and female subjects. Additionally, the study primarily utilized SHR rats as an ADHD model, which, while widely accepted, does not fully recapitulate the heterogeneity of ADHD observed in humans. Future investigations should incorporate additional animal models and clinical studies to validate the translational potential of BSKQF.

NF-κB plays a dual role in ADHD pathogenesis, regulating both microglia-mediated neuroinflammation and synaptic plasticity as well as blood-brain barrier integrity [[49], [50], [51]]. We assessed NF-κB pathway activation in microglia under OGD/R conditions. NF-κB, a key nuclear transcription factor, is upregulated in activated microglia and plays a central role in inflammation. Upon activation, phosphorylation of IκBα leads to its dissociation from the NF-κB-IκB complex, allowing p65 phosphorylation and nuclear translocation to initiate transcription of inflammatory mediators such as IL-6 [[52], [53], [54]]. BSKQF treatment inhibited OGD/R-induced NF-κB-p65 activation, with the most pronounced effect observed at the medium dose. Interestingly, this result differs from in vivo observations, possibly due to immune tolerance mechanisms in the physiological environment. Furthermore, BSKQF also suppressed NLRP3 inflammasome activation, showing a trend consistent with NF-κB-p65 inhibition. Notably, all BSKQF serum concentrations reduced TNF-α production, with the high-dose group exerting superior suppression compared to the medium-dose group. This discrepancy suggests that high-concentration BSKQF may enhance anti-inflammatory effects via additional pathways.

In summary, our study confirms that SHR rats exhibit ADHD-like behavioral characteristics and excessive microglial activation. These findings provide new insights into the regulatory effects of BSKQF on ADHD and highlight potential novel therapeutic targets for its treatment.

## CRediT authorship contribution statement

**Jiaqi Zhang:** Writing – review & editing, Writing – original draft, Supervision, Methodology, Formal analysis, Data curation, Conceptualization. **Ruxin Sun:** Writing – review & editing, Supervision, Methodology, Investigation, Data curation. **Yu Xiong:** Writing – review & editing, Supervision, Methodology, Investigation, Data curation. **Yuting Yang:** Writing – review & editing, Supervision, Investigation, Formal analysis. **Jing Wang:** Methodology, Investigation. **Kanglin Zhu:** Writing – review & editing, Validation, Supervision, Data curation. **Xinqiang Ni:** Writing – review & editing, Validation, Resources, Project administration, Methodology, Funding acquisition.

## Ethics approval and consent to participate

The animal experiment was approved by the Laboratory Animal Ethics Committee of and conducted in strict accordance with the Guide for the Care and Use of Laboratory Animals issued by the National Institutes of Health. The animal experiment was approved by the Laboratory Animal Ethics Committee of Guangzhou University of Chinese Medicine (Approval No.20230221109) and conducted in strict accordance with the Guide for the Care and Use of Laboratory Animals issued by the National Institutes of Health and the ARRIVE guidelines. Male SHRs were used in this study to minimize the potential variability associated with estrous cycle in females.

## Consent for publication

Not applicable.

## Availability of data and materials

All data generated or analyzed during this study are included in this published article. Further inquiries can be directed to the corresponding authors.

## Clinical trial number

Not applicable.

## Funding

This research was funded by 10.13039/501100001809National Natural Science Foundation of China (No. 82074492, 82374517), Project of Commission on Innovation and Technology of Shenzhen (JCYJ20230807094808017).

## Declaration of competing interest

The authors declare that they have no known competing financial interests or personal relationships that could have appeared to influence the work reported in this paper.

## Data Availability

No data was used for the research described in the article.

## Abbreviations

ADHD: Attention Deficit Hyperactivity Disorder
BSKQF: Bushen Kaiqiao Formula
TCM: Traditional Chinese Medicine
SHR: Spontaneously Hypertensive Rats
WKY: Wistar Kyoto Rats
UHPLC-MS/MS: Ultra-high-performance liquid chromatography coupled with a tandem mass spectrometer
ELISA: Enzyme-Linked Immunosorbent Assay
OGD/R: Oxygen-Glucose Deprivation/Reoxygenation
NF-κB: Nuclear Factor kappa-light-chain-enhancer of activated B cells
NLRP3: NOD-like Receptor Protein 3
TMRE: Tetramethylrhodamine, ethyl ester
IL-1β: Interleukin 1 beta
IL-6: Interleukin 6
TNF-α: Tumor Necrosis Factor alpha
iNOS: inducible Nitric Oxide Synthase
Iba-1: Ionized calcium-binding adaptor molecule 1
PPI: Protein–Protein Interaction
GO: Gene Ontology
KEGG: Kyoto Encyclopedia of Genes and Genomes
TEM: Transmission Electron Microscopy
EPM: Elevated Plus Maze
OFT: Open Field Test
CMC-Na: Sodium Carboxymethyl Cellulose
DAPI: 4′,6-diamidino-2-phenylindole
FBS: Fetal Bovine Serum
PBS: Phosphate Buffered Saline
RIPA: Radio-Immunoprecipitation Assay
HRP: Horseradish Peroxidase
SDS-PAGE: Sodium Dodecyl Sulfate Polyacrylamide Gel Electrophoresis
PVDF: Polyvinylidene Difluoride
ECL: Enhanced Chemiluminescence
OD: Optical Density
